# Clinical and laboratory evaluation of a real-time PCR for *Clostridium difficile* toxin A and B genes

**DOI:** 10.1007/s10096-012-1558-1

**Published:** 2012-02-14

**Authors:** E. de Jong, A. S. de Jong, C. J. M. Bartels, C. van der Rijt-van den Biggelaar, W. J. G. Melchers, P. D. J. Sturm

**Affiliations:** Department of Medical Microbiology, Radboud University Nijmegen Medical Centre, P.O. Box 9101, 6500 HB Nijmegen, Netherlands

## Abstract

Many laboratories use enzyme immunoassays (EIAs) for the diagnosis of *Clostridium difficile* infection (CDI). More recently, polymerase chain reaction (PCR)-based diagnosis has been described as a sensitive test. Real-time PCR for the detection of *C. difficile* toxin A and B genes was evaluated. A prospective evaluation was performed on stool samples from 150 hospitalized adult patients and 141 healthy volunteers. PCR was compared to toxigenic culture (TC), direct cytotoxicity test (CTT), ImmunoCard® Toxin A and B (Meridian Bioscience), and enzyme-linked immunosorbent assay (ELISA) (Vidas). The results were correlated with clinical data using a standardized questionnaire. The diagnostic yield of the PCR was further evaluated after implementation. Using toxigenic culture as the gold standard, the sensitivity and specificity of PCR were 100 and 99.2%, respectively. Patients were categorized as follows: TC/PCR-positive (*n* = 17) and negative TC (*n* = 133). The differences in these groups were more frequent use of antibiotics and leukocytosis (*p* < 0.05). The diagnostic yield of PCR was evaluated during a period of 6 months and showed an increase of positive patients by 50%. PCR for the detection of toxigenic *C. difficile* has a high sensitivity and can rule out CDI, but cannot differentiate CDI from asymptomatic carriage. Clinicians should be aware of this in order to prevent inappropriate treatment and delay of other diagnostics.

## Introduction

The laboratory diagnosis of *Clostridium difficile* infection (CDI) is based on the demonstration of toxin A/B directly in stool samples or in culture after isolation of the pathogen. The direct cytotoxicity test (CTT) has been the gold standard in laboratory diagnosis; more recently, toxigenic culture (TC) has been used for this purpose [1]. Both methods are not suitable for routine diagnosis since results are delayed (at least to more than 24 h), they are labor-intensive, and fresh cell cultures are needed. To overcome these problems, numerous enzyme immunoassays (EIAs) have been developed which are now used widely in clinical laboratories [2]. However, the described performance of EIAs varies widely, with sensitivity and specificity ranging from 23 to 99% and 70 to 100%, respectively [3–9]. More recently, polymerase chain reaction (PCR) has been studied in the diagnosis of CDI [8, 10–13]. A number of studies demonstrate that PCR is a sensitive diagnostic test. The reported specificities range between 94 and 99.2%, which may result from the lower sensitivity of the gold standard, a common problem in the evaluation of the highly sensitive PCR-based methods. Furthermore, asymptomatic carriage of toxigenic *C. difficile* has been described in 0.5–13% of adults and may be of concern to the use of PCR.

In the current prospective study, PCR was compared to TC, CTT, and two EIAs for the routine diagnosis of CDI. In contrast to published PCR studies, our test results were correlated to clinical presentation and follow-up. To estimate the potential problem of asymptomatic carriage, PCR was performed in healthy volunteers.

## Materials and methods

### Setting and specimens

The study was performed in a tertiary teaching hospital with approximately 30,000 patient admissions annually. Initial PCR evaluation was performed on 45 culture-positive stool samples. Thereafter, consecutive stool specimens that were submitted to the routine clinical microbiology laboratory for *C. difficile* toxin detection were collected prospectively from 150 hospitalized adult patients. Only one specimen per patient was included. No outbreaks of diarrheal pathogens were recorded by the hospital infection control unit of our hospital in the study period.

Upon receipt at the laboratory, the routinely used diagnostic ImmunoCard® Toxin A and B test (ICTAB) was performed and four aliquots of the stool specimen were stored at −80°C for subsequent testing in batch by TC, PCR, CTT, and enzyme-linked immunosorbent assay (ELISA).

To correlate test results with clinical presentation, clinical data on signs and symptoms were collected. To assess the occurrence of asymptomatic carriage, healthy adult volunteers were recruited among medical students and hospital employees. Volunteers were informed on the purpose of the study and that the results would not be reported. Exclusion criteria for volunteers were the use of antibiotics or complaints of diarrhea. All specimens were thawed only once before testing in batch. Specimens from all volunteers were tested by TC and PCR, and positive specimens were further tested using ICTAB, ELISA, and CTT.

The study was approved by the hospital ethics committee.

### Routinely used enzyme immunoassay (ICTAB)

The routinely used diagnostic ICTAB test ImmunoCard® (Meridian Bioscience, Cincinnati, OH, USA) was performed following the manufacturer’s instructions. The results were interpreted independently by two technicians.

### Toxigenic Culture (TC)

*C. difficile* selective agar with cefoxitin, amphotericin B and cycloserine (CLO, bioMérieux), and Columbia blood agar with colistin and nalidixic acid (CAP, Oxoid, Cambridge, UK) were inoculated with 10 μl of the stool specimen. The media were incubated for 5 days under anaerobic conditions at 37°C. Colonies with growth characteristics of *C. difficile* were investigated by sequence analysis of the 16S rRNA for identification [14]. *C. difficile* isolates were subcultured in brain heart infusion (BHI, CM0225, Oxoid) for the detection of toxin production using the cytotoxicity test and PCR detecting genes encoding for toxin A and B.

Ribotyping was performed on all isolates by the National Reference Laboratory for *Clostridium difficile*.

### Direct cytotoxicity test (CTT)

The CTT was used to determine the presence of toxin in stool samples (direct CTT) and toxin production in *C. difficile* isolates (TC).

The supernatant of 1 mg stool suspended in 1 ml phosphate buffered saline (PBS) was filtered through a 0.45-μM filter (Millipore®, Billerica, MA, USA). Quantities of 20 μl of filtrate and 20 μl of filtrate with 20 μl of anti-*C. sordellii* antitoxin (SAT, Uniprom, T5000, Krimpen aan de IJssel, the Netherlands) were incubated on Vero cells for 48 h at 37°C. Appropriate controls were included with each micro-titer plate used, i.e., 20 μl of toxin (*C. difficile* Bess strain), 20 μl of SAT, and 20 μl toxin with 20 μl SAT. The specimen was positive if 50% of the cells showed a characteristic cytopathic effect (cell rounding) which was neutralized by SAT.

The cytotoxicity of the isolates was tested as described above using a 25-μl aliquot of BHI after filtering through a 0.45-μM filter.

### PCR

DNA extraction: 200 μl supernatant of a 10% fecal suspension in PBS was used for DNA isolation on MagNA Pure LC using the MagNA Pure LC Total Nucleic Acid Isolation Kit (Roche Diagnostics, Almere, the Netherlands). For PCR on cultured bacteria, a single colony was boiled for 10 min in 50 μl of glycerol broths. Quantities of 5 μl of the prepared DNA were used for the PCR reactions.

Real-time PCR assay: a triplex real-time PCR assay was developed to simultaneously detect the *C. difficile tcdA* and *tcdB* toxin genes and the *gB* polymerase gene of phocine herpes virus (PhHV-1), which served as the internal control [15]. A positive PCR result did not distinguish between the presence of the toxin A and/or B gene; *tcdA* primers and probes were adapted from Bélanger et al.[16], i.e., the anti-sense molecular beacon was replaced by a sense TaqMan probe (5′-CTACTAgAggAAgAgATTCAAAATCCTCA-3′); *tcdB* and PhHV primers and probes were as described [12, 15]. PCR mixes consisted of 25 μl of 2 × LC480 Probes Master (Roche Diagnostics, Almere, the Netherlands), 0.5 μM of each primer and 0.1 μM of each probe, and 5 μl of extracted DNA. Real-time PCR was performed on a Roche LC480.

DNA sequencing for confirmation of the identification of *C. difficile*: 16S rRNA gene sequences were determined using primers described by Weisburg et al. [14]. All DNA sequences were determined using a BigDye Terminator version v3.1 cycle sequencing kit (ABI) and an ABI 3100 DNA sequencer.

### ELISA

The VIDAS CDAB test (biomérieux, Marcy-l’Etoile, France), an ELISA for the detection of toxin A and B, was performed according to the manufacturer’s instructions. All stool samples were tested in batch by one technician.

### Clinical evaluation

Demographic data and laboratory results were collected from the electronic patient files. Patients’ medical history and current signs and symptoms were collected by interviewing the attending physician and reviewing the medical charts using a standardized questionnaire. Data collected included reason for and duration of admission, characteristics of the current episode of diarrhea (consistency, frequency, duration, and abdominal pain), C-reactive protein (CRP) and white blood cell count (WBC), use of antibiotics in the previous 3 months, and the presence of other risk factors for *C. difficile* infection (prior hospitalization, use of immunosuppressive medication, proton pump inhibitors, gastric tube feeding, chemotherapy, abdominal surgery).

The clinical course was evaluated retrospectively in patients who were negative by ICTAB but were positive by PCR in order to investigate the clinical significance of these positive results.

### Diagnostic yield after implementation of the PCR

The results of *C. difficile* detection in routine clinical specimens were extracted from the laboratory information system for the first 6 months after implementation of the PCR and compared to the results obtained in the 6 months prior to implementation of the PCR when laboratory diagnosis was based toxin detection using ICTAB. All PCR-positive specimens were cultured as described above.

### Statistical analysis

The Chi-squared test and Fisher’s exact test were used to analyze the significance of clinical parameters.

## Results

All of the 45 culture-positive stool samples from the initial evaluation were positive on PCR testing.

A total of 150 patients were included during a 2-month period, of which 49.7% were male and the median age was 61 years (range 19–95). Most patients were admitted to the medical wards (56%), followed by the surgical (20.7%) and hematology/oncology wards (20.7%) and the intensive care units (2.6%).

Using the TC as the gold standard, the sensitivity, specificity, positive predictive value (PPV), and negative predictive value (NPV) of the PCR, ICTAB, ELISA, and CTT are shown in Table 1.

**Table 1:** Sensitivity, specificity, positive predictive value (PPV), and negative predictive value (NPV) using toxigenic culture (TC) as the gold standard

	Sensitivity	Specificity	PPV	NPV
Polymerase chain reaction (PCR)	100%	99.2%	94.4%	100%
Direct cytotoxicity test (CTT)	70%	100%	100%	96.3%
ImmunoCard® Toxin A and B (ICTAB)	47%	99.2%	88.9%	93.6%
Enzyme-linked immunosorbent assay (ELISA)	58.8%	89.4%	62.5%	96.7%

TC was positive in 17 patients (11%). PCR was positive in 18 patients, including all 17 TC-positive patients, as well as one patient with negative results for all other tests and repeated negative stool cultures. CTT was positive in 12 patients, all of whom had a positive TC. The routinely used ICTAB was positive in nine patients; eight of these patients had a positive TC. The ELISA was positive in 16 patients, but only ten of these had a positive TC. Equivocal ELISA results were found in five patients, and three of these had a positive TC. Respectively, 5, 9, and 4 patients with a positive TC were negative using CTT, ICTAB, and ELISA. An overview of all the test results is presented in Table 2.

**Table 2:** Test results for the different tests in 150 patients

Number of patients	TC	PCR	CTT	ICTAB	ELISA
7	+	+	+	+	+
2	+	+	+	−	+
3	+	+	+	−	Eq
1	+	+	−	−	+
3	+	+	−	−	−
1	+	+	−	+	−
1	−	+	−	−	−
1	−	−	−	+	−
6	−	−	−	−	+
2	−	−	−	−	Eq
123^a^	−	−	−	−	−

^a^Four patients had a non-toxigenic strain

Eq: equivocal on repeated testing

The isolates belonged to the following ribotypes: ICTAB-positive: 001, 011, 014, 018, 053, 056, and 078; ICTAB-negative: 001, 002, 014, 046, 079, 165, and two unknown. Two ICTAB-negative but TC- and PCR-positive specimens contained the ribotypes known to cause false-negative ICTAB results.

According to the laboratory findings, the patients were categorized in patients with a positive TC (*n* = 17), of which five had a negative CTT, and a negative TC (*n* = 133). Patient characteristics and clinical data were compared in these patient categories (Table 3). More prior antibiotic use and a higher occurrence of elevated WBC was present in patients with a positive TC (*p* < 0.05). Within the group of patients with positive TC, no differences were found between patients with positive (*n* = 12) and negative CTT (*n* = 5); however, the number of patients was low. There was only one patient with a positive PCR only.

**Table 3:** Patient characteristics and clinical data of patients with positive and negative TC

	TC-positive (*n* = 17)	TC-negative (*n* = 133)^a^
Fever >38.5°C	5/16 (31%)	50/127 (39%)
Diarrhea		
>48 h	9/17 (53%)	64/133 (49%)
>24 h	2/17 (11%)	23/133 (17%)
No	6/17 (36%)	46/133 (34%)
Abdominal pain	7/16 (44%)	33/127 (26%)
Antibiotics		
In prior 3 months	16/17 (94%)^b^	84/131 (64%)^b^
No	1/17 (6%)	47/131 (36%)
Gastric tube feeding	3/17 (18%)	27/133 (20%)
Chemotherapy	2/17 (12%)	21/133 (16%)
Prior CDI	2/17 (12%)	5/133 (4%)
Proton pump inhibitor	8/17 (47%)	50/133 (38%)
Immunocompromised	7/17 (42%)	62/133 (47%)
Ward		
Medical	8/17 (47%)	76/133 (57%)
Surgical	6/17 (35%)	25/133 (19%)
ICU	0/17	4/133 (3%)
Hematology/oncology	3/17 (18%)	28/133 (21%)
WBC		
>15 × 10^9^/l	6/17 (35%)^b^	14/133 (10%)^b^
<15 × 10^9^/l	9/17 (53%)	110/133 (83%)
Unknown	2/17 (12%)	9/133 (7%)
CRP		
<5 mg/l	3/17 (18%)	11/133 (8%)
>15 mg/l	10/17 (59%)	83/133 (62%)
5–15 mg/l	1/17 (6%)	17/133 (13%)
Unknown	3/17 (18%)	22/133 (17%)

CDI: *Clostridium difficile* infection; ICU: intensive care unit; WBC: white blood cell count; CRP: C-reactive protein

^a^Including one patient with a positive PCR only

^b^ *p* < 0.05

The median cycling time (CT) value of PCR was 26.44 (range 21.58–34.69) for CTT-positive stool samples, 38.15 (range 25.51–38.36) for CTT-negative stool samples, and 36.32 for the TC/CTT-negative stool sample.

The clinical course was investigated retrospectively in ten patients who were not diagnosed by our routinely used ICTAB (Table 4), i.e., nine patients with positive TC and PCR, and one patient with a positive PCR only. Five of these patients also had a positive CTT. One of these patients (patient 5, Table 4) was diagnosed with pseudomembranous colitis (PMC) by endoscopic evaluation, and one patient (patient 2) was clinically diagnosed with CDI despite the negative laboratory result. Both patients recovered with metronidazole therapy. One patient (patient 1) recovered after the treatment of graft-versus-host disease (GVHD), which was diagnosed by the histological examination of colon biopsies, and in the two other patients (patients 3 and 4), symptoms resolved within 2 days without treatment, despite the continuation of antibiotic therapy.

**Table 4:** Follow-up of patients with negative ImmunoCard® Toxin A and B but who were positive by TC

PCR-positive, CTT-positive, TC-positive	Sex and age (years)	Admission	Risk factors	Infectious diarrhea	CRP (mg/l)	WBC (×10^9^/ml)	Diagnosis	Origin
Patient 1	F, 43	Vomiting, diarrhea	Use of antibiotics, proton pump inhibitors (PPI), prior CDI, immunocompromised	*Salmonella*, *Shigella*, and *Campylobacter* (SSC)-negative	<5	4.3	Graft-versus-host disease (GVHD), clinical response on GVHD therapy	HA
Patient 2	F, 55	Diarrhea, abdominal pain	Immunocompromised, use of antibiotics	SSC-negative	7	15.2	Diverticulosis, suspicion of CDI, and treatment with metronidazole	HA
Patient 3	F, 80	Pneumonia	Chemotherapy, PPI, use of antibiotics	Not tested	20	7.3	Spontaneous recovery without therapy	HA
Patient 4	M, 39	Abdominal surgery	Abdominal surgery	Viral PCR-negative	Unknown	Unknown	Spontaneous recovery without therapy	HA
Patient 5	F, 86	Diarrhea	Use of antibiotics	SSC-negative	130	38.6	Pseudomembranous colitis (PMC) histologically proven, treated with metronidazole	HA
PCR-positive, CTT-negative, TC-positive								
Patient 6	M, 51	GVHD	Immunocompromised, use of antibiotics	SSC-, rotavirus-, and adenovirus-negative	52	8.3	GVHD, died of respiratory failure	HA
Patient 7	F, 93	Diarrhea, dehydration	Use of antibiotics	SSC-negative	144	9.7	Slow spontaneous recovery	Nursing home
Patient 8	M, 24	Pneumonia	Immunocompromised, PPI, use of antibiotics	SSC- and viral PCR-negative	32	23.7	Spontaneous recovery during therapy	Home
Patient 9	M, 58	Malaise	PPI, use of antibiotics	Not tested	9	11.5	Spontaneous recovery without therapy	HA
PCR+, CTT-, TC-								
Patient 10	M, 72	Dyspnea	Abdominal surgery, prior CDI, PPI, use of antibiotics	SSC-negative	200	10.5	Spontaneous recovery	HA

HA: healthcare-associated within 3 months of prior hospitalization; M: male; F: female

Four patients had a positive TC and PCR but negative CTT. One patient (patient 6) was diagnosed with GVHD by the histology of colon biopsies and died because of extensive disease. One patient (patient 7) had severe diarrhea and recovered from recurrent episodes of diarrhea in a timescale of several months without intervention. One patient (patient 8) recovered within two days without intervention. One patient (patient 9) was admitted with diarrhea after antibiotic treatment and recovered with calcium supplements for hemodialysis treatment. The patient with a positive PCR (patient 10) only developed recurrent episodes of diarrhea during multiple treatment courses with antibiotics and recovered each time after the cessation of treatment.

To estimate the potential problem of asymptomatic carriage, PCR was performed in healthy volunteers. During a 2-month period, specimens were submitted by 141 unique volunteers. Of these, 30.5% were male, and the median age was 25 years (range 18–76). Most volunteers were students (49%), followed by non-medical hospital employees (27%), laboratory technicians (17%), and physicians (7%). TC, PCR, and CTT were positive in 1 (0.7%) volunteer, and ICTAB was negative.

In our laboratory, unformed stool specimens are tested for *C. difficile* toxin at the doctor’s request and if the patient has been admitted for 72 h or more. After implementation of the PCR in the routine laboratory, the percentage of patients with positive results for *C. difficile* increased by more than 50%. From 1 January 2010 until 30 June 2010, before implementation of the PCR, 895 stool specimens from 694 patients were tested and 7.3% of the specimens and 8.6% (60) of the patients were positive for *C. difficile* using ICTAB only. From 1 January 2011 until 30 June 2011, after implementation of the PCR, 809 stool specimens from 657 patients were tested and 12.7% of the specimens and 13.5% (89) of the patients were positive for *C. difficile* using the PCR only. Two of the 103 PCR-positive specimens were not available for culture. Of the remaining 101 specimens, all were confirmed by culture except for one.

## Discussion

PCR is a sensitive and specific method for the detection of toxin-producing *C. difficile*. In this study, the sensitivity and specificity were 100 and 99%, respectively, using TC as the gold standard, which is in keeping with previous studies [1]. CTT was positive in only 70% of the specimens with a positive TC and the EIAs used had a sensitivity of 47 and 58.8%. The specificity of CTT, ICTAB, and ELISA were 100, 99, and 89%, respectively. The low sensitivity of ICTAB could not be ascribed to the presence of difficult-to-detect ribotypes as described previously; only 2 of the 9 false-negative were type 002 [17].

The interpretation of a positive TC and/or PCR result is difficult since asymptomatic carriage of *C. difficile* occurs frequently. In our study, TC/PCR as well as CTT was positive in only one of 141 healthy volunteers (0.7%). Other studies have detected asymptomatic carriage of toxigenic *C. difficile* in healthy subjects of between 0.5 and 13% [18, 19]. Moreover, in hospitalized patients, carriage has been described in up to 14% [20, 21]. Therefore, the mere demonstration of *C. difficile* capable of producing toxin A and/or B does not necessarily mean that it is the causative agent of the clinical signs and symptoms in a patient [20].

Taking this relatively high occurrence of asymptomatic carriage into consideration, one could hypothesize that the demonstration of toxin activity directly in the stool by CTT may be more predictive for the presence of CDI. However, the CTT was also positive in the one healthy volunteer in this study, and in a study by Kyne et al., 79% of 19 asymptomatic carriers had a positive CTT directly in stool [20].

The median CT value was higher in the patients with a negative CTT compared to the patients who were TC-, PCR-, and CTT-positive. However, in this small series, there was considerable overlap of CTs. It, therefore, does not seem to be helpful to determine the clinical significance in the individual patient.

In addition to the fact that laboratory tests cannot distinguish between infection and colonization, in many cases, a clinical diagnosis of CDI is complicated because hospitalized patients with diarrhea due to *C. difficile* share signs and symptoms with diarrhea due to other causes, especially antibiotic treatment itself. Indeed, in the patients included in this study, who were already identified to be at risk for CDI by the clinician, there were no significant differences between patients with and without a positive result for *C. difficile* with respect to clinical presentation, except for more prior antibiotic use and a higher WBC in patients with positive results. Even in retrospect, a clinical diagnosis of CDI was difficult to establish. Since the routine diagnosis of *C. difficile* in our laboratory was based solely on the ICTAB results and the other tests were done later in batch, we identified nine additional patients with positive TC, of whom five had a positive CTT and one patient with a negative TC and positive PCR. Review of their medical records showed that, in one patient with a positive CTT, a diagnosis of CDI was established by endoscopy and another patient with a positive CTT was successfully treated for CDI despite the negative routine results. On the other hand, symptoms resolved within 2 days in two patients with a positive CTT despite the continuation of antibiotics and one patient had a diagnosis of GVHD. Thus, except in one patient, CDI could not be proven, but it certainly could not be ruled out either since CDI may be self-limiting. Even the patient with an alternative diagnosis of GVHD may have had concurrent CDI. In the five patients who were positive by PCR and negative by CTT (one was also TC-negative), a clinical diagnosis of CDI could also not be established in retrospect: one was diagnosed with extensive GVHD and the other four patients recovered without intervention.

Community-acquired CDI and CDI in the absence of a history of antibiotic use are increasingly reported and will further complicate a clinical diagnosis of CDI in the future [22].

After substitution of ICTAB by the PCR in the routine clinical laboratory, the percentage of positive patients increased by more than 50%, which confirms the high sensitivity of the PCR described in this study in a larger number of patients with 809 specimens from 657 patients tested, of which 103 specimens were positive. The specificity for the presence of toxigenic *C. difficile* in the stool was also confirmed, as 100 of the 101 PCR-positive specimens were confirmed by TC.

In conclusion, PCR for the detection of toxigenic *C. difficile* has a high sensitivity and can rule out CDI, but it cannot differentiate CDI from asymptomatic carriage. CTT does not seem to differentiate between infection and carriage either. Clinicians should be aware of the occurrence of asymptomatic carriage to prevent inappropriate administration of antibiotics or cessation of antibiotic therapy, and to prevent delay of other diagnostics or therapy.
